# Non-invasively recorded transient pathological high-frequency oscillations in the epileptic brain: a novel signature of seizure evolution

**DOI:** 10.1186/1471-2202-16-S1-P32

**Published:** 2015-12-18

**Authors:** Catherine Stamoulis, Bernard Chang

**Affiliations:** 1 Harvard Medical School, Boston, MA, 02115, USA; 2 Departments of Radiology and Neurology, Boston Children's Hospital, Boston, MA, 02115, USA; 3 Department of Neurology, Beth Israel Deaconess Medical Center, Boston, MA, 02215, USA

## Background

Brain activity is characterized by oscillatory patterns that occur at a wide range of frequencies and have been associated with various cognitive processes and behaviors. Depending on their dominant frequency, brain oscillations facilitate the coordinated activation of neuronal networks in response to external inputs and cognitive demands. The vast majority of human studies have focused on neural oscillations at frequencies typically less than 50 Hz. In contrast, relatively few studies have investigated neural activity above 50-60 Hz, in part due to the fact that relatively low-amplitude oscillations at these frequencies are difficult to identify in human electrophysiological signals, particularly from the intact brain. Both physiological and pathological oscillations at frequencies >80 Hz have been previously reported, but their occurrence, dynamics and role remain elusive. Pathological high-frequency oscillations (HFO) detected in invasive recordings from the epileptic brain may be correlated with epileptogenic brain tissue and may provide insights into the physiology of the hyperexcitable brain. While recent studies have shown that HFOs are also detectable in scalp electroencephalography (EEG) [1], very little is known about their physiological origin and relationship to seizure dynamics.

## Methods

This study investigated the dynamics of scalp-recorded transient high-frequency oscillations (sHFO) identified in continuous multi-day EEG recordings from 12 epilepsy patients with medically intractable seizures. Using a time-domain decomposition approach for non-stationary signals, individual EEG signals were decomposed into their dominant components, including low-amplitude non-random, non-artifact signals with characteristic frequencies >80 Hz. Based on their frequency and waveform durations these corresponded to transient sHFOs.

## Results

Transient, low-amplitude (typically <20 μV) oscillations with durations of <50 - ~100 ms and dominant frequencies in the range >80-190 Hz were identified in continuous scalp EEG in both wakefulness and sleep. Based on their frequencies these oscillations fell into two distinct ranges ~80-125 Hz and ~150-195 Hz, and occurred intermittently over long periods of time (of the order of several hours). Seizures consistently occurred during intervals of increased sHFO amplitude and decreased sHFO frequency and were often time-locked to local changes in sHFO parameters. Finally, sHFOs were found to be spatially localized both within and beyond the epileptogenic region, with asymmetric spatial patterns that were more localized in interictal, immediate preictal and postictal intervals and more spatially distributed during ictal epochs, possibly associated with seizure propagation.

## Conclusions

Low-amplitude sHFOs were consistently detected in continuous EEG often hours prior to ictal onset. These oscillations had short durations, their temporal patterns were independent of the sleep-wake cycle, and were detected primarily in electrodes covering the epileptogenic region as well as a few contralateral electrodes. The majority of seizures occurred during intervals of increased sHFO amplitude, suggesting that these oscillations may be associated with a relatively long period of ictogenesis. Consequently, sHFOs may represent a novel electrophysiological signature of the epileptic brain, and may be associated with local generators of abnormal neural activity beyond the epileptogenic region. These findings further suggest that sHFO may dynamics may be used for targeted clinical intervention to prevent seizure occurrence.
